# Trends in the Prevalence of Diarrheagenic *Escherichia coli* among Hospitalized Diarrheal Patients in Kolkata, India

**DOI:** 10.1371/journal.pone.0056068

**Published:** 2013-02-14

**Authors:** Sanjucta Dutta, Sucharita Guin, Santanu Ghosh, Gururaja P. Pazhani, Krishnan Rajendran, Mihir K. Bhattacharya, Yoshifumi Takeda, G. Balakrish Nair, Thandavarayan Ramamurthy

**Affiliations:** 1 Division of Bacteriology, National, Collaborative Research Centre of Okayama University for Infectious Diseases in India, West Bengal, India; 2 Clinical Division, Institute of Cholera and Enteric Diseases, Collaborative Research Centre of Okayama University for Infectious Diseases in India, West Bengal, India; 3 National Institute of Cholera and Enteric Diseases, Kolkata, West Bengal, India; 4 Translational Health Science and Technology Institute, Gurgaon, Haryana, India; University College Dublin, Ireland

## Abstract

**Background:**

To analyse the trends in the prevalence of different pathogroups of diarrheagenic *Escherichia coli* (DEC) among hospitalized acute diarrheal patients.

**Methodology/Principal Findings:**

From the active surveillance of diarrheal disease at the Infectious Diseases Hospital, Kolkata, 3826 **s**tool specimens collected during 2008–2011 were screened for DEC and other enteric pathogens. PCR was used in the detection of enterotoxigenic, enteropathogenic and enteroaggregative *E. coli* and 10 major colonization factor antigens (CFs) of enterotoxigenic *E. coli*. The relationship between DEC infected patient’s age group and clinical symptoms were also investigated. Multiplex PCR assay showed that the prevalence of EAEC was most common (5.7%) followed by ETEC (4.2%) and EPEC (1.8%). In diarrheal children >2 year of age, EAEC and EPEC were detected significantly (*p* = 0.000 and 0.007, respectively). In children >2 to 5 and >5 to 14 years, ETEC was significantly associated with diarrhea (*p* = 0.000 each). EAEC was significantly associated with diarrheal patients with age groups >14 to 30 and >30 to 50 years (*p* = 0.001, and p = 0.009, respectively). Clinical symptoms such as vomiting, abdominal pain, watery diarrhea, were recorded in patients infected with ETEC. Dehydration status was severe among patients infected by ST-ETEC (19%) and EPEC (15%). CS6 was frequently detected (37%) among ETEC.

**Conclusions/Significance:**

Hospital based surveillance reviled that specific pathogroups of DEC are important to certain age groups and among ETEC, CS6 was predominant.

## Introduction

Diarrhea and other related gastrointestinal disorders are the most important causes of illness and death, particularly among infants and young children from developing countries [1]. Of the 4.879 million global deaths of children below 5 years of age due to infectious diseases, diarrhea alone has caused 0.801 million deaths in 2010. During the same period, diarrheal deaths in India were 0.212 million [1]. Diarrhea can be caused by bacterial, viral and parasitic pathogens. *Escherichia coli* is one of the members of the family Enterobacteriaceae, which resides as a commensal flora in the intestinal lumen of animals and humans. Among children below five years of age, diarrheagenic *E. coli* (DEC) such as enterotoxigenic *E. coli* (ETEC), enteropathogenic *E. coli* (EPEC), enteroaggregative *E. coli* (EAEC) are the most important enteric pathogens and are responsible for 30 to 40% of all the diarrheal episodes in developing countries [2], [3].

ETEC has two major virulence associated factors, the heat-stable (ST) and/or heat-labile (LT) enterotoxins, and several antigenic fimbriae known as colonization factors (CFs) [4]. EPEC generates attaching and effacing (AE) lesions and harbor *eae,* encoding the structural gene for intimin, which is located the locus for enterocyte effacement [5]. EPEC is classified into typical (tEPEC) and atypical (aEPEC) strains based on the presence of the plasmid *E. coli* adherence factor (EAF). EAEC is associated with persistent diarrhea in children and has the ability to adhere to Hp2 cell line in a stacked brick configuration termed aggregative adherence (AA). The AA phenotype is associated with specific fimbriae (AAFs) encoded by plasmids (pAAs) [6]. A cryptic sequence known as CVD432 in the pAA has been used as an EAEC molecular marker in several epidemiological studies [7]. The pAA carries a transcription activator encoding gene *aggR*, which is designated as a major EAEC virulence regulator. The EAEC strains that harbor the AggR regulon are the true or typical EAEC [8]. AggR initiates the expression of chromosomally located contiguous genes including the *aai* that encodes for AggR-activated Island [9].

In this study, we investigated the prevalence of DEC and the types of CFs in ETEC from acute diarrheal patients admitted at the Infectious Diseases Hospital (IDH), Kolkata using culture and PCR assays. We also analysed the distribution of DEC among different age groups and sole infections status to understand its nature of infection with respective to the clinical outcome of the patients.

## Methods

### Clinical Specimens

From January 2008 to December 2011, 3826 stool specimens collected from acute diarrheal patients hospitalized at the IDH were tested in this study. The enrolled patients belonging to any age group/sex represent every fifth hospitalised case with diarrhea on two randomly selected days in a week. Passage of three or more loose stools in 24 hrs, with or without clinical symptoms of an enteric ailment (nausea, vomiting, abdominal pain or cramps, dehydration, fecal urgency, or dysentery), was considered as diarrhea. Stool specimens were collected using sterile catheters in McCartney bottles and examined for its consistency and other characteristics including the presence of blood/mucus. These specimens were examined within 2 hrs in the laboratory for common enteric pathogens using the published methods [10], [11].

### Isolation and Identification of DEC

For isolation of *E. coli*, stool specimens were plated on MacConkey (Difco, Sparks Glencoe, MD, USA) followed by incubation for 16–18 hrs at 37°C. Three typical lactose fermenting pink colour colonies per sample were selected and subcultured in Luria Bertani agar (LBA, Difco) plates. Cultures from this non-selective medium were tested for indole-production by scraping a small portion of culture on a sterile cotton swab and dropping of the Kovacs reagent. Change of this swab tips to pink colour was recorded positive and further tested in triple sugar iron agar for typical *E. coli* biochemical reactions.

### Detection of DEC Virulence Genes by PCR

### Preparation of DNA Templates for PCR

A small portion of indole positive bacterial growth was taken from each of the three colonies plated on LBA plates and mixed with 500 µl of phosphate-buffer saline in 1.5 ml microfuge tubes and boiled for 10 min in a water bath followed by snap chilling in ice for 5 min. The heat-treated bacterial suspensions were centrifuged at 10,000 rpm for 5 min to pellet down the cell debris and the supernatants were used as DNA templates in the PCR.

### Multiplex PCR Assay

The DNA templates were subjected to multiplex PCR with specific primers for the detection of virulence marker genes such as *eae, bfpA* (structural genes of EPEC), *eltB* and *estA* (enterotoxin genes of ETEC), CVD432 (the nucleotide sequence of the *Eco*RI-*Pst*I DNA fragment of pCVD432 of EAEC) [11], [12] and *aaiC* (encodes a secreted protein of the EAEC pathogenicity island AAI, which is co-ordinately regulated by the AggR activator). The ETEC positive strains were also screened for the 10 major CFs as described previously [13].

PCRs was performed with a 20-µl reaction mixture containing 3 µl of template DNA, 2.5 µl of 10× PCR buffer II, 2 µl of a 1.25 mM mixture of deoxynucleoside triphosphates, 0.25 µl of 5 U of Taq-polymerase per µl (New England Biolabs, USA), and a 20 mM of each primer (Sigma, Bangalore, India). The cycling conditions in a GeneAmp PCR system 9700 (AB Applied Biosystem) were as follows: 96°C for 4 min, 95°C for 20 sec, 57°C for 20 sec, and 72°C for 1 min in 35 cycles, with a final 7-min extension at 72°C. PCR products (10 µl) were confirmed by electrophoresis using a 2% (wt/vol) agarose gel (Sigma). The DNA bands were visualized and photographed under UV light after the gel was stained with ethidium bromide.

In this study, we adopted the published protocols for the detection of other enteric pathogens including the enteric viruses [10], [11].

### Statistical Analysis

The age of the patients were classified into 4 categories viz., the all age groups, <2, >2–5, >5–14 and >14 years. The categorized DEC was compared with each age group along with other pathogens detected in this study. Fisher's exact test was performed to establish mutual relatedness among the three types of DEC pathogroups. *P* values of <0.05 were considered as statistically significant and calculated the odds ratio (OR) and the 95% confidence interval (95% CI).

### Ethics Statement

Ethical approval was obtained from the National Institute of Cholera and Enteric Diseases Ethics Committee (Ref.C-4/2012-T&E), and each participant/parent in the case of children gave written informed consent.

## Results

During 2008–2011, 3826 patients admitted at the IDH for the treatment of diarrhea were enrolled in this study. Considering the selection strategy, this figure represents a total of more than 19,000 diarrheal patients who received treatment after hospitalization. DEC was detected in 11.8% (452/3826) of the patients from whom 30.5% (138/452) and 69.5% (314/452) were identified as sole and mixed pathogens, respectively (Table 1). The other pathogens such as vibrios, salmonellea, shigellae, campylobacters, aeromonads, enteric parasites and viruses were detected in 2265 (59.2%) stools specimens whereas in 1109 (29%) there was no known etiological agents.

**Table 1:** Prevalence of DEC pathogroups among different age groups of diarrheal patients.

DEC	All Age group			Age ≤2 years				>2–5 years			>5–14 years			>14 years		
	Total	Sole	Mixed	Total	Sole		Mixed	Total	Sole	Mixed	Total	Sole	Mixed	Total	Sole	Mixed
ETEC LT	34 (0.9)	16 (0.4)	18 (0.5)	9 (1)		1(0.1)	8 (0.9)				1 (0.4)		1 (0.4)	24 (1)	15 (0.6)	9 (0.4)
ETEC ST	68 (1.8)	31 (0.8)	37 (1)	8 (0.9)		1 (0.1)	7 (0.8)	5 (2.1)	3 (1.2)	2 (0.8)	3 (1.1)		3 (1.1)	52 (2.1)	27 (1.1)	25 (1)
ETEC LT+ST	62 (1.6)	24 (0.6)	38 (1)	10 (1.2)		1 (0.1)	9 (1)				2 (0.7)	2 (0.7)		50 (2)	21 (0.9)	29 (1.2)
EAEC	218 (5.7)	48 (1.3)	170 (4.4)	94 (10.9)		16 (1.9)	78 (9)	18 (7.5)		18 (7.5)	12 (4.3)	5 (1.8)	7 (2.5)	94 (3.9)	27 (1.1)	67 (2.7)
aEPEC	48 (1.2)	13 (0.3)	35 (0.9)	12 (1.4)		1 (0.1)	11 (1.3)	2 (0.8)	1 (0.4)	1 (0.4)	4 (1.4)	2 (0.7)	2 (0.7)	30 (1.3)	9 (0.4)	21 (0.8)
tEPEC	22 (0.6)	6 (0.2)	16 (0.4)	13 (1.5)		3 (0.4)	10 (1.1)	3 (1.2)	1 (0.4)	2 (0.8)				6 (0.2)	2 (0.1)	4 (0.2)
Nopathogen	1109 (29)			110 (12.8)				47 (19.5)			75 (26.7)			877 (35.9)		
Total	3826			863				241			281			2441		

Figures in parentheses indicate %.

The patients were categorized into 4 age groups viz ≤2, >2–5, >5–14 and >14 years and checked for predominance of specific pathogroups of DEC if any. Overall, the detection rate of DEC was more in children ≤2 (16%, 146/863) than >14 years age groups (10.5%, 256/2441) (Table 1). Among the DEC positive samples, the detection rate of EAEC, ETEC and EPEC was 48.2% (218/452), 36.3% (164/452) and 15.5% (70/452), respectively. In majority of the ETEC infected cases, strains harboring the virulence genes *est* or with *est* and *elt* (68 and 62 cases, respectively) were more compared to the strains that harbors *elt* alone (34 cases). Of the 70 EPEC, 22 and 48 were identified as tEPEC and aEPEC, respectively (Table 1). Atypical EPEC was relatively high in >5 years age group. Among EAEC, pCVD432 was detected in 83 strains and the *aaiC* harboring strains alone or with pCVD432 were detected in 91 and 44 strains, respectively. The proportion of *aaiC* alone or with pCVD432 was uniformly high in patients who had mixed infection (∼81%) than the sole infection caused by the EAEC (∼18%).

The DEC was predominantly associated with other pathogens as the sole infection rate was comparatively less in all the age groups. The isolation rate of DEC was higher in 2009 compared to other years (data not shown). In addition, there was no well-marked seasonal trend in the prevalence of any particular pathogroup of DEC. In order to identify the preponderance of any DEC to specific age group of the patients, we made a multinomial regression analysis (Table 2). We used >50 year age group as a reference to explore the high age risk group to the specific DEC pathogroup. In this model, EAEC and EPEC were isolated significantly among children <2 years of age (Table 2; Odds Ratio = 5.87, 95% confidence interval (CI) 3.46–9.98, *p* = 0.000 and 3.00, 95% CI 1.35–6.68, *p* = 0.007, respectively). In children >2 to 5 and >5 to 14 years ETEC was significantly detected (Table 2; Odds Ratio  = 0.13, 95% CI 0.05–0.34, *p* = 0.000, and 0.16, 95% CI0.07–0.38, *p* = 0.000, respectively). In >14 to 30 and >30 to 50 year age group EAEC was significantly detected (Table 2; Odds Ratio = 2.69, 95% CI 1.51–4.77, *p* = 0.001, and 2.19, 95% CI 1.21–3.95, *p* = 0.009, respectively).

**Table 2:** Multinomial logistic regression models exploring significant risk age group of predominant DEC infection at IDH, Kolkata.

Age groupin years	DEC pathogroup	B	OR (95% CI)	*p*-value
**> = 2**	ETEC	−0.43	0.65(0.38–1.08)	0.099
	EPEC	1.09	3.00(1.35–6.68)	0.007*
	EAEC	1.77	5.87(3.46–9.98)	0.000*
**>2–5**	ETEC	−2.00	0.13(0.05–0.34)	0.000*
	EPEC	−0.47	0.62(0.20–1.91)	0.410
	EAEC	0.12	1.12(0.57–2.21)	0.732
**>5–14**	ETEC	−1.82	0.16(0.07–0.38)	0.000*
	EPEC	−0.69	0.50(0.15–1.66)	0.258
	EAEC	−0.29	0.75(0.35–0.58)	0.451
**>14–30**	ETEC	0.08	1.08(0.69–1.69)	0.733
	EPEC	0.40	1.50(0.61–3.67)	0.374
	EAEC	0.99	2.69(1.51–4.77)	0.001*
**>30–50**	ETEC	0.28	1.32(0.86–2.03)	0.197
	EPEC	0.63	1.87(0.79–4.42)	0.151
	EAEC	0.78	2.19(1.21–3.95)	0.009*
**>50**		Reference category		
		

* Statistically significant.

Comparison of different clinical symptoms caused by the various pathogroups of DEC indicated that vomiting was predominant (>75%) among EPEC, ST or LT and ST producing ETEC infected cases (Table 3). More than 50% of the DEC positive patients had watery diarrhea and the proportion was high (>70%) among the patients who were identified with ST, LT and ST-producing ETEC (Table 3). Abdominal pain was the other common symptom generally observed in >35% of the DEC infected cases (Table 3). Severe dehydration was the other symptom detected among patients infected with EPEC and ST-producing ETEC (>15%).

**Table 3:** Clinical symptoms of patients with sole infections caused by different pathogroups of DEC.

Clinical symptoms	EAEC 48/218	ETEC-ST 31/68	ETEC-LT 16/34	ETEC LT+ST 24/62	EPEC 19/70
Vomiting	29 (60.4)	24 (77.4)	8 (50.0)	20 (83.3)	16 (84.2)
Fever	2 (4.2)	4 (12.9)	2 (12.5)	1 (4.2)	4 (21.1)
Abdominal pain	17 (35.4)	15 (48.4)	7 (43.8)	10 (41.7)	7 (36.8)
Type of diarrhoea					
Bloody	1 (2.1)				
Watery	33 (68.8)	22 (71.0)	11 (68.8)	21 (87.5)	10 (52.6)
Semisolid	14 (29.2)	9 (29.0)	5 (31.3)	3 (12.5)	9 (47.4)
Dehydration status					
Severe	3 (6.3)	6 (19.4)	1 (6.3)	1 (4.2)	3 (15.8)
Some	44 (91.7)	25 (80.6)	15 (93.8)	23 (95.8)	15 (78.9)
None	1 (2.1)				1 (5.3)

Numbers in parentheses indicates %. Numerators are number of patients with sole (single DEC) pathogen.

and the denominators represent numbers of patients infected with DEC along with other pathogen(s).

All the 163 ETEC strains were screened for the major CFs such as CFAI, CFAIII, CS1 to CS6, CS14 and CS17 by PCR. We identified the CFs only in 52 ETEC strains (32%) and rest of the 112 strains were negative for the specific CFs targeted in this study. CFAI, CS6 were detected in higher proportion in ST-producing ETEC, whereas CFAIII and CS5, were exclusively found in LT-producing ETEC (Table 4). Of the 52 ETEC that were positive in CFs, majority of the strains produced both LT and ST (56%).

**Table 4:** Prevalence of CFs among different types of ETEC.

CF type	ETEC-ST	ETEC-LT	ETEC LT+ST	Total
CFAI	6	1	3	10
CFAIII		1		1
CS1	1	1		2
CS3		2	5	7
CS4	1			1
CS5		2		2
CS6	3	1	4	8
CS14	1			1
CS17			3	3
CS1+CS3			2	2
CS2+CS3			6	6
CS5+CS6		3	6	9

## Discussion

Among the hospitalized diarrheal children up to 5 years of age, DEC was high next to rotavirus and in patients more than 5 years; DEC-mediated diarrheal infection was positioned next to cholera [10]. Prevalence of DEC in this study was almost 12% which is more than the other reports from developing countries [14], [15]. Prevalence of different pathogroups of DEC was not uniform in all age groups. Significant association of EAEC and EPEC were detected in children ≥2 year of age. Similar trend was reported in Tanzania in children 0–6 months of age [16]. Our study also detected ETEC in age groups from >2 to 14 years and EAEC with the age group>14 years. Several reports indicate a strong association of ETEC in younger children [14], [17] and EAEC in higher age groups [18]. DEC-mediated diarrhea is not specific to any season and found throughout the year. In other studies conducted in developing countries showed a strong seasonality of DEC associated infection [19]–[22]. Watery diarrhea, vomiting are the important clinical symptoms associated with sole infections caused by many of the DEC. The dehydration status was severe in cases which were positive for *est* harboring ETEC as well as EPEC strains.

Prevalence of EAEC has been high compared to other reports [19]–[21]. In addition to the CVD432 marker the chromosomal marker virulence gene *aaiC* was included in the screening. With these two gene markers, the identification rate of EAEC was more compared to a previous study conducted in Kolkata [23]. Interestingly, in many strains we have identified *aaiC* gene that were devoid of the CVD432 virulence marker. The *aaiC* or *aggR* positive *E. coli* strains are increasingly detected from patients presenting with diarrhea [24].

As seen in this study, the detection rate of ETEC was higher compared to other reports [19]–[21]. Infections caused by *elt* harboring ETEC strains was less (8%) compared to ETEC with *est* alone and combination with *elt* (14% each). World-wide, *est* was found to be associated with the severe outcome of the disease [25]–[28]. With the PCR based assay, we could detect only 32% of the CFs in this study which is much low compared to a monoclonal antibody-based dot blot based study conducted in Bangladesh [29]. Similar to an early report, ETEC harboring *est* predominantly had the genes encoding for CFAI and CS6 [30]. However, CF profile with *elt* harboring ETEC strains seems to be unique in this region.

Prevalence of EPEC in this study was less compared to other investigations conducted in developing countries. The rate of isolation of the EPEC was much higher in Chile (38.3%) and Brazil (34.0%) whereas the frequency was lowest in Somalia (4.0%) and Thailand (5.5%) [31]–[33]. Atypical EPEC has been found more t than tEPEC in both developing and developed countries [33]. In the present study, prevalence of typical and atypical EPECs remained almost the same in age groups ≤2 and >2–5 years. Studies conducted in many countries indicated that tEPEC was more in infants <6 months old [33]. In other age groups, aEPEC predominated. Considering its world-wide occurrence as an etiological agent of diarrhea, the aEPEC has been considered as one of the emerging pathogens in developing and developed countries [34].

The reason for higher prevalence of aEPEC is not clear. Many findings indicate that aEPEC may have an innate property of persisting longer in the intestine than the other pathogroups of DEC. Due to the presence of *bfpA*, the tEPEC adhere firmly to the epithelial cells and disrupts normal cellular process [33], [35]. On the other hand, the aEPEC decreases apoptosis of intestinal epithelial cells thereby favouring protracted intestinal colonization.

This study suggests that DEC is one of the important pathogens that should be considered in the surveillance programme of diarrheal diseases. aEPEC is involved in large number of diarrheal cases in and it may be an emerging pathogen. Further studies are needed to investigate the epidemiology and virulence properties of atypical EPEC strains.
